# Host-dependent editing of SARS-CoV-2 in COVID-19 patients

**DOI:** 10.1080/22221751.2021.1969868

**Published:** 2021-09-05

**Authors:** Josep Gregori, Maria Francesca Cortese, Maria Piñana, Carolina Campos, Damir Garcia-Cehic, Cristina Andrés, Josep Francesc Abril, Maria Gema Codina, Ariadna Rando, Juliana Esperalba, Elena Sulleiro, Joan Joseph, Narcís Saubí, Sergi Colomer-Castell, Mari Carmen Martin, Carla Castillo, Juan Ignacio Esteban, Tomas Pumarola, Francisco Rodriguez-Frias, Andrés Antón, Josep Quer

**Affiliations:** a Liver Diseases-Viral Hepatitis, Liver Unit, Vall d’Hebron Institut de Recerca (VHIR), Vall d’Hebron Barcelona Hospital Campus, Barcelona, Spain; b Centro de Investigación Biomédica en Red de Enfermedades Hepáticas y Digestivas (CIBERehd), Instituto de Salud Carlos III, Madrid, Spain; c Roche Diagnostics SL, Barcelona, Spain; d Biochemistry and Microbiology Departments, Vall d’Hebron Institut de Recerca (VHIR), Vall d’Hebron Barcelona Hospital Campus, Barcelona, Spain; e Respiratory Viruses Unit, Microbiology Department, Vall d’Hebron Institut de Recerca (VHIR), Vall d’Hebron Barcelona Hospital Campus, Barcelona, Spain; f Computational Genomics Lab, Genetics, Microbiology and Statistics Department, Institut de Biomedicina (IBUB), Universitat de Barcelona, Barcelona, Spain; g Microbiology Department, Vall d’Hebron Hospital Universitari, Vall d’Hebron Barcelona Hospital Campus, Barcelona, Spain; h Universitat Autònoma de Barcelona, Bellaterra, Spain

**Keywords:** ADAR1, SARS-CoV-2, editing, mutations, quasispecies

## Abstract

A common trait among RNA viruses is their high capability to acquire genetic variability due to viral and host mechanisms. Next-generation sequencing (NGS) analysis enables the deep study of the viral quasispecies in samples from infected individuals. In this study, the viral quasispecies complexity and single nucleotide polymorphisms of the SARS-CoV-2 *spike* gene of coronavirus disease 2019 (COVID-19) patients with mild or severe disease were investigated using next-generation sequencing (Illumina platform). SARS-CoV-2 *spike* variability was higher in patients with long-lasting infection. Most substitutions found were present at frequencies lower than 1%, and had an A → G or T → C pattern, consistent with variants caused by adenosine deaminase acting on RNA-1 (ADAR1). ADAR1 affected a small fraction of replicating genomes, but produced multiple, mainly non-synonymous mutations. ADAR1 editing during replication rather than the RNA-dependent RNA polymerase (nsp12) was the predominant mechanism generating SARS-CoV-2 genetic variability. However, the mutations produced are not fixed in the infected human population, suggesting that ADAR1 may have an antiviral role, whereas nsp12-induced mutations occurring in patients with high viremia and persistent infection are the main source of new SARS-CoV-2 variants.

## Introduction

Severe acute respiratory syndrome coronavirus 2 (SARS-CoV-2), the causal agent of the acute respiratory syndrome known as coronavirus disease 2019 (COVID-19), replicates by using its own RNA-dependent RNA polymerase (nsp12). SARS-CoV-2 expresses an accessory non-structural protein, nsp14, which has 3’-5’ exonuclease proofreading activity. This feature, which notably limits the acquisition of mutations during replication of the viral genome, is seen in other coronaviruses, such as SARS-CoV and mouse hepatitis virus [1, 2]. Phylogenetic analysis of SARS-CoV-2 consensus sequences has shown only small differences [3], but other sources of variation (insertions, deletions, and recombination events) have been described in several coronaviruses, including SARS-CoV-2 [4–10].

Animal cells have a first-line innate immunity that enables a prompt response to danger signals and pathogens. Interactions between the specialized cellular receptors known as pattern recognition receptors (PRRs) and conserved pathogen-associated molecular patterns (PAMPs) rapidly trigger an innate intracellular antiviral response, usually based on immediate activation of the interferon and NF-kb pathways [11]. Unlike bacteria, viral DNA and RNA have certain features (unusual in the cellular host) that can directly activate specific PRRs. One such PRR is adenosine deaminase acting on RNA1 (ADAR1). ADAR1 directly attacks the virus by deaminating adenosine (A) in double-stranded RNA, thereby producing inosine (I) which is misinterpreted as guanosine (G) by the splicing and translation machinery. ADAR1 editing of double-stranded RNA (dsRNA) can be specific (deamination of selected A residues) or almost random. In long dsRNA (>100 bp), this editing can affect 50% of all adenosine residues and cause a hypermutation, as has been reported in some single-stranded RNA viruses during replication and persistent infection [12]. In contrast, short dsRNSs (∼20–30 bp) and long dsRNAs with bulges, helixes, hairpins, and loops are edited selectively and only a few adenosines are specifically chosen [13].

RNA editing by ADAR1 has been reported in several viruses [14], where it may contribute to inhibiting viral infection [15]. However, viruses can also take advantage of the variability introduced by cellular mutagenic enzymes, including ADAR1, to support viral infectivity and evolution [16–18]. Adenosine deamination in codon 196 of the hepatitis delta virus (HDV) antigenome enables the expression of large delta antigen, which has a key role in viral replication [19]. In human immunodeficiency virus 1 (HIV-1), measles virus (MV), vesicular stomatitis virus (VSV), and hepatitis D virus (HDV), ADAR1 enhances replication during the acute phase of infection [20]. It acts by editing the viral substrates – adding A to G substitutions to the RNA genome [21] – or by inhibiting dsRNA-dependent protein kinase (PKR) [20]. Due to the large genome size of SARS-CoV-2 (≈30 kb) and its complex tridimensional structure [22], the ADAR enzyme together with other deaminases may be involved in the genome editing process [23].

SARS-CoV-2 has shown low variability to date, but deep analysis of the viral genome might detect variants overlooked by traditional sequencing. We hypothesized that in-depth analysis of the SARS-CoV-2 *spike* gene in samples obtained from COVID-19 patients at the beginning of the pandemic could reveal the underlying mechanisms causing variants in this part of the genome before they become fixed in the viral quasispecies. Are the substitutions, present at low levels, due to replication errors produced by the viral polymerase or to interactions with enzymes inherent to the patients’ immune system? In addition, examination of the viral quasispecies in patients with different clinical profiles (mild or severe disease) could reveal clinical parallels. Finally, after determining the mechanisms that generate replication errors, it could be of interest to compare the mutations found with those recurrently detected worldwide after several months of the pandemic to study the impact of these mechanisms on the profile of fixed mutations.

In this study, we analyzed the SARS-CoV-2 *spike* gene by next-generation sequencing (NGS) in samples obtained from COVID-19 patients. Our aim was to meticulously determine the presence of nucleotide mutations in the viral genome and investigate the contribution of the intracellular mutagenic enzyme, ADAR1, to the variability observed.

## Patients and methods

### Patients

The study included samples from 18 patients with mild or severe COVID-19 admitted to the emergency room of Hospital Universitari Vall d’Hebron (HUVH), Barcelona (Spain) in March 2020. SARS-CoV-2 infection was diagnosed in the HUVH Microbiology Department using two tests, an in-house PCR assay using the primer/probe set from the CDC 2019-nCoV real-time RT-PCR diagnostic panel (Qiagen, Germany) and the commercial Allplex 2019-nCoV real-time RT-PCR assay (Seegene, Korea). Samples had been collected from the upper respiratory tract (nasopharyngeal/oropharyngeal swabs or nasopharyngeal aspirates). Ten patients had mild symptoms (absence of influenza-like illness or hypoxia and no hospitalization requirement), and 8 patients had severe disease (e.g. pneumonia, hypoxemic respiratory failure, sepsis, cardiomyopathy and arrhythmia, acute kidney dysfunction) and were admitted to the intensive care unit. All patients had a favourable outcome, with resolution of the infection (Table 1). No viral or bacterial co-infections were reported.

**Table 1.** Clinical and demographic characteristics of COVID-19 patients included in the study.

	MILD (*n* = 10)	Severe (*n* = 8)
*Sample type*		
Nasopharyngeal aspirate	30% (3/10)	12.5% (1/8)
Nasopharyngeal/oropharyngeal SWAB	70% (7/10)	87.5% (7/8)
RT-PCR threshold, Ct median[IQR]*	23.4 [19.6; 25.5]*	30.8 [24.2; 32.7]*
Sex, % men	30% (3/10)	50% (4/8)
Age, years	42 [28; 50]*	48 [45; 50]*
Days in intensive care unit	Not admitted	16 [7; 25]*

The asterisk (*) indicates the minimum and maximum value of the intervals included in each square bracket. Ct, cycle threshold; IQR, interquartile range.

The patients included had no comorbidities other than acute respiratory syndrome and were all SARS-CoV-2 monoinfected. All cases included occurred during the first wave of the COVID-19 pandemic, in which treatment of severely ill patients consisted mainly of respiratory maintenance because there had been no previous experience in the management of these patients.

### Methods

#### SARS-CoV-2 sequencing and quality control

Viruses were inactivated by mixing respiratory specimens with AVL buffer (Qiagen, Hilden, Germany). Viral RNA was extracted using the QIAmp Viral RNA Mini Kit (Qiagen, Hilden, Germany) following the manufacturer’s instructions. The complete *spike* gene was amplified and sequenced into 13 overlapping amplicons, as previously reported by Andres et al. [10].

The RT-PCR amplification and sequencing method has been described [10]. The final library was loaded in a MiSeq Reagent Kit 600V3 cartridge (Illumina, San Diego, CA) and sequenced using the MiSeq platform (Illumina, San Diego, CA), which, in our experience, has the highest resolution power [24, 25]. To obtain high-quality haplotypes, the sequencing reads underwent rigorous quality filters [26]. Reads had to have a minimum of 20 overlapping bases and a maximum of 10% mismatches. Reads that did not fulfil these requirements and those with more than 1% of bases below a Phred score of Q33 (0.9995 accuracy) were excluded.

Reads were demultiplexed by matching primers, allowing a maximum of three mismatches, and the primer sequences at both ends were trimmed off. Identical reads were collapsed to haplotypes, and their corresponding frequencies were expressed as read counts. A fasta file was generated with each pool/primer/strand combination. Reverse haplotypes were reverse-complemented. Haplotypes with more than three gaps were removed. Remaining gaps were repaired based on the most abundant haplotype (master) in the corresponding quasispecies. Haplotypes were selected only if they had both a forward and reverse strand and were present at >0.1% in each strand, providing a robust quality control.

To compensate for the differing coverage of the samples, we established a reference coverage of 70,000 reads and an abundance threshold of 0.15%. Haplotypes present at ≤0.15% were excluded [27].

All computations were done in the R language and platform [28], and in-house scripts were developed using Biostrings [29], Ape [30], ShortRead [31], and ggseqlogo [32].

#### Quasispecies diversity

Quasispecies diversity indices were calculated based on the characteristics and frequency of all haplotypes observed: number of haplotypes per amplicon, number of polymorphic sites per amplicon, frequency of the dominant haplotype per amplicon, rare haplotype load (RHL) at 0.1% and at 1% per amplicon, Shannon entropy, and mutation frequency [27, 33–36].

#### Analysis of variants

Minority variants were identified per amplicon, haplotype, and site, and were categorized by type and frequency. Overlapping regions were used as controls to compare variants identified in consecutive amplicons. Potential bias effects due to the primers were controlled by inspecting how the forward and reverse primers of each amplicon were seen in the previous and posterior amplicons, thanks to the amplicon overlapping. No significant conflicts were observed.

Haplotypes in each sample/amplicon were right trimmed to the next amplicon and the *spike* gene was analyzed haplotype-by-haplotype and site-by-site. A variant was defined as a nucleotide that differed from that of the corresponding dominant haplotype (master sequence).

To study eventual substitution biases, the frequency of a variant per site was computed as the number of haplotypes bearing the variant in the overall study population, without taking into consideration the haplotype frequency in the viral quasispecies of each specific sample/amplicon. The Fisher test was used to contrast biases in the base substitutions from the expected values, and to determine homogeneity vs independence in the distribution of pairs of substitutions.

The above-described analyses were conducted for both the overall study population and for each specific patient. The frequency of each nucleotide, the number of polymorphic sites, and the substitution type (transition or transversion) were determined for each patient.

#### Characterization of single nucleotide variants (SNVs) by type and frequency

The 3’ end of each amplicon was trimmed to avoid redundancies in the overlaps, and the alignment was used to count the substitutions that occurred. Regardless of the abundance of each haplotype in the quasispecies, the number of SNVs was computed with respect to the quasispecies dominant haplotype in each sample/amplicon.

SNVs were characterized by substitution type and by frequency. Variant abundance was expressed as the percentage of each SNV within the particular quasispecies (per patient and amplicon). To avoid redundancy, SNV percentages in overlapping regions of the amplicon were averaged. In addition, SNVs were characterized as the frequency per abundance bin; that is, the number of SNVs in all the samples (per patient and amplicon) with abundances in the following bins: (0%, 0.2%], (0.2%, 0.5%], (0.5%, 1%], (1%, 2%], (2%, 5%], (5%,10%], and (10%,50%]. The lower bins would be representative of the underlying mechanism causing the substitutions, whereas the higher bins would incorporate the effects of haplotype fitness and selection.

#### Location of substitutions in the *spike* gene secondary structure

To study how SNVs could affect the secondary structure of SARS-CoV-2 RNA in the *spike* region, we used the Wuhan-Hu-1 (MN908927.2) *spike* gene sequence, with the D614G (A23403G) substitution in the S protein as the backbone, into which each naturally observed nucleotide substitution was inserted. A minimum free energy (MFE) secondary structure of the *spike* gene (positive sense for A > G and negative sense for T > C) was generated for each mutant using a loop-based energy model and a dynamic programming algorithm [37], and the RNA folding tools offered by the University of Vienna website resources [38, 39].

#### Characterization of fixed mutations worldwide

To study the type of mutations fixed in SARS-CoV-2 isolates worldwide, we extracted the main SNVs per position from the 353,341 events observed in 48,625 GISAID genomes by Mercatelli and Giorgi [40]. Mutations supported by at least 10 genomes were considered the main SNVs. This computation eliminated prevalence effects due to bias in the geographic origins, resulting from the very different number of genomes from different places. The aim of this computation was not to identify the most prevalent SNVs, but instead, to identify any SNV observed in any genome worldwide and to obtain a count of mutation types.

## Results

### *Spike* gene quasispecies diversity

The SARS-CoV-2 *spike* gene was extracted, amplified, and sequenced in all patients. In total, 41,326,097 reads passed our quality filter, with a median coverage of 145,391 reads per amplicon (interquartile range [IQR] 115,576–201,833). Coverage of amplicons from patients with severe disease tended to be higher than that of patients with a mild condition (Table 2).

In total, 1536 haplotypes were detected overall with no large differences between the two clinical groups: a median of 4 and 3.5 haplotypes per amplicon was detected in mild and severe cases, respectively (Supplementary Table S1).

Most haplotypes per amplicon (1130/1536, 73.6%) were present at ≤0.5% in the respective quasispecies (Supplementary Table S2). In general, each haplotype had a single mutation relative to the master haplotype (Supplementary Table S3).

Analysis of the quasispecies diversity indices in relation to COVID-19 severity showed that diversity (rare haplotype load at 1%, Shannon entropy, and mutation frequency) tended to be higher in severe than in mild cases (Table 2). This trend could be related to the effects of severe disease, to the higher viral load, or to the lengthier disease duration in severe cases. However, some patients with mild disease showed high diversity, whereas some with severe disease showed low diversity (Supplementary Tables S1–S4) with no correlations with viral load, indicating that higher diversity may be driven by the duration of the infection.

**Table 2.** Statistics of diversity indices regarding the haplotype characteristics and abundance within the corresponding quasispecies.

Index	Condition	Min	1st *Q*	Median	Mean	3rd *Q*	Max
Depth	Severe	89,820	132,475	192,410	214,754	267,141	520,239
	Mild	71,306	105,405	127,254	146,090	165,103	492,886
# Hpl	Severe	1	2	4	7.82	10.25	32
	Mild	1	1	4	5.56	7	24
# PolySites	Severe	0	1	3	6.73	9.25	31
	Mild	0	0	3	4.58	6.75	23
% Master	Severe	73.4	96.1	98.9	96.7	99.6	100
	Mild	91.9	97.9	99.3	98.6	100	100
RHL@1%	Severe	8.19%	11.78%	13.55%	14.76%	16.04%	28.24%
	Mild	8.0%	11.0%	12.91%	13.63%	15.27%	22.85%
RHL@0.1%	Severe	7.64%	10.47%	11.66%	11.72%	12.85%	17.17%
	Mild	7.23%	10.03%	11.18%	11.34%	12.41%	16.40%
Shannon	Severe	0	0.0263	0.0740	0.1830	0.2592	11,727
	Mild	0	0	0.0512	0.0938	0.1402	0.4859
Mf × 10^−6^	Severe	0	9.28	30.44	96.65	112.93	659.67
	Mild	0	0	24.17	41.79	59.99	264.10

Depth, number of reads per amplicon; # Hpl, number of haplotypes per amplicon; Mf, mutation frequency per amplicon; # % Master, percentage of the most abundant haplotype in the amplicon (master sequence); PolySites, number of polymorphic sites per amplicon; RHL@1%, rare haplotype load at 1%; RHL@0.1%, rare haplotype load at 0.1%; Shannon, Shannon entropy.

### *Spike* gene variants

Comparison of *spike* consensus sequences from all patients with sequences from the Wuhan reference genome (MN908947.3) found that all patients but one (P02) had the A1841G substitution (D614G), which classifies the virus as clade G, and two other patients (P07 and P17) had the G3707T (C1236F) and G3231A (syn.) substitution, respectively.

### Minority variants

**Figure 1.**
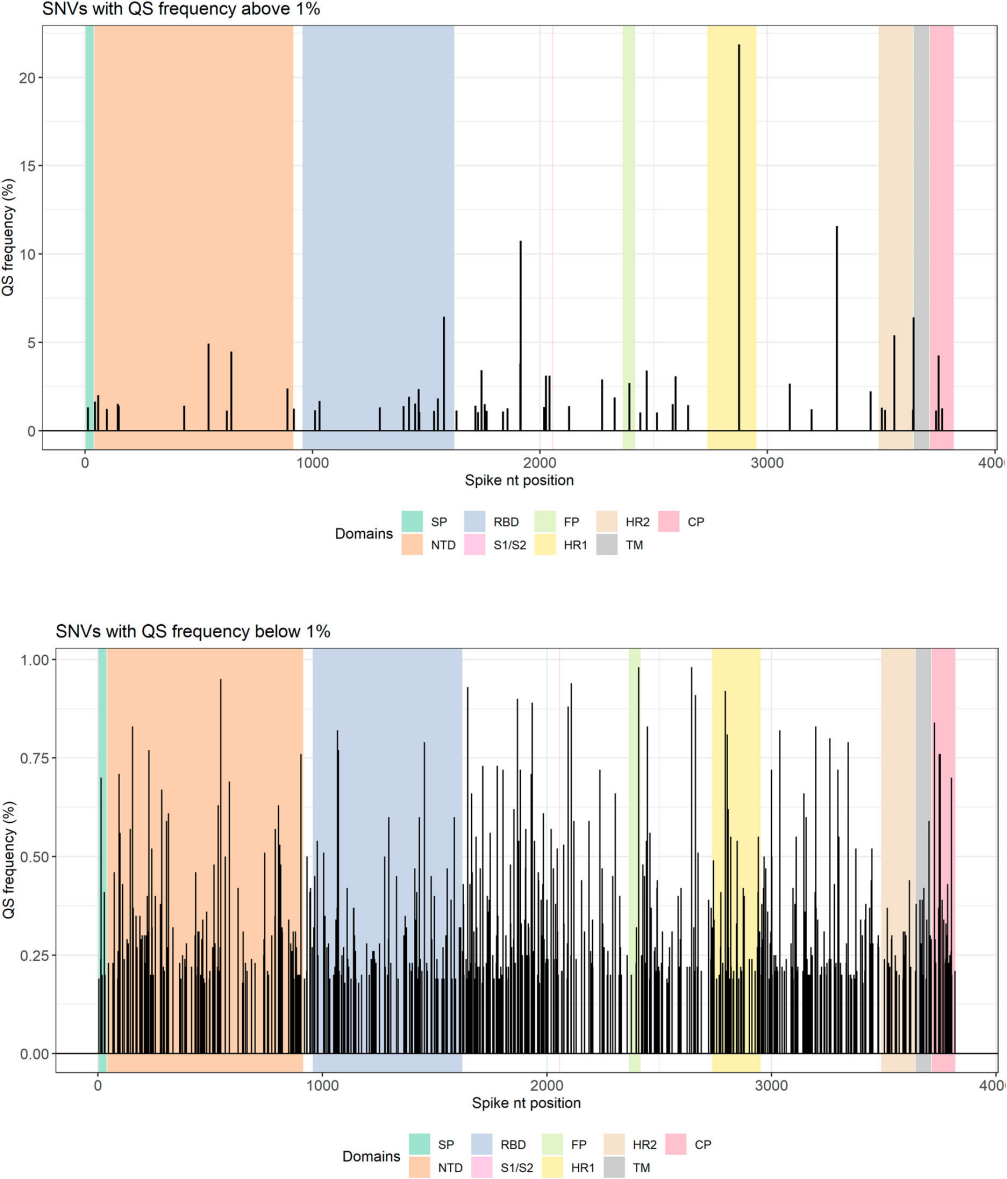
Single nucleotide variants (SNVs) according to their frequency and position in the *spike* (S) region. Top panel shows SNVs with frequencies above 1%. Bottom panel shows SNVs below 1%. The *spike* regions are depicted in different background colours. CP, cytoplasmic domain; FP, fusion peptide; HR, heptad repeat; NTD, N-terminal domain; QS, quasispecies; RBD, receptor-binding domain; SP, signal peptide; TM, transmembrane.

In the total of patients, variant frequency was lower than 2% in most patients with mild disease except for C0890T (S0297L) in P14 and C1467T (Syn) in P15, present at 2.4% and 2.35%, respectively (Supplementary Tables S4 and S5). Variants accounting for >5% of the viral quasispecies were seen in three patients with severe disease (Figure 1 and Supplementary Table S4). In two of the three, variants were present at >10%: T2875C (Syn) at 21.85% in P08, and G3306T (W1102C) at 11.6% and G1915T (G639C) at 10.8% in P11. The majority of highly frequent mutants (>2% of quasispecies) were found in severe cases (P08, P09, P10, P11, P12, P13, P16, P17) except for the two mutations mentioned above in P14 and P15.

**Figure 2.**
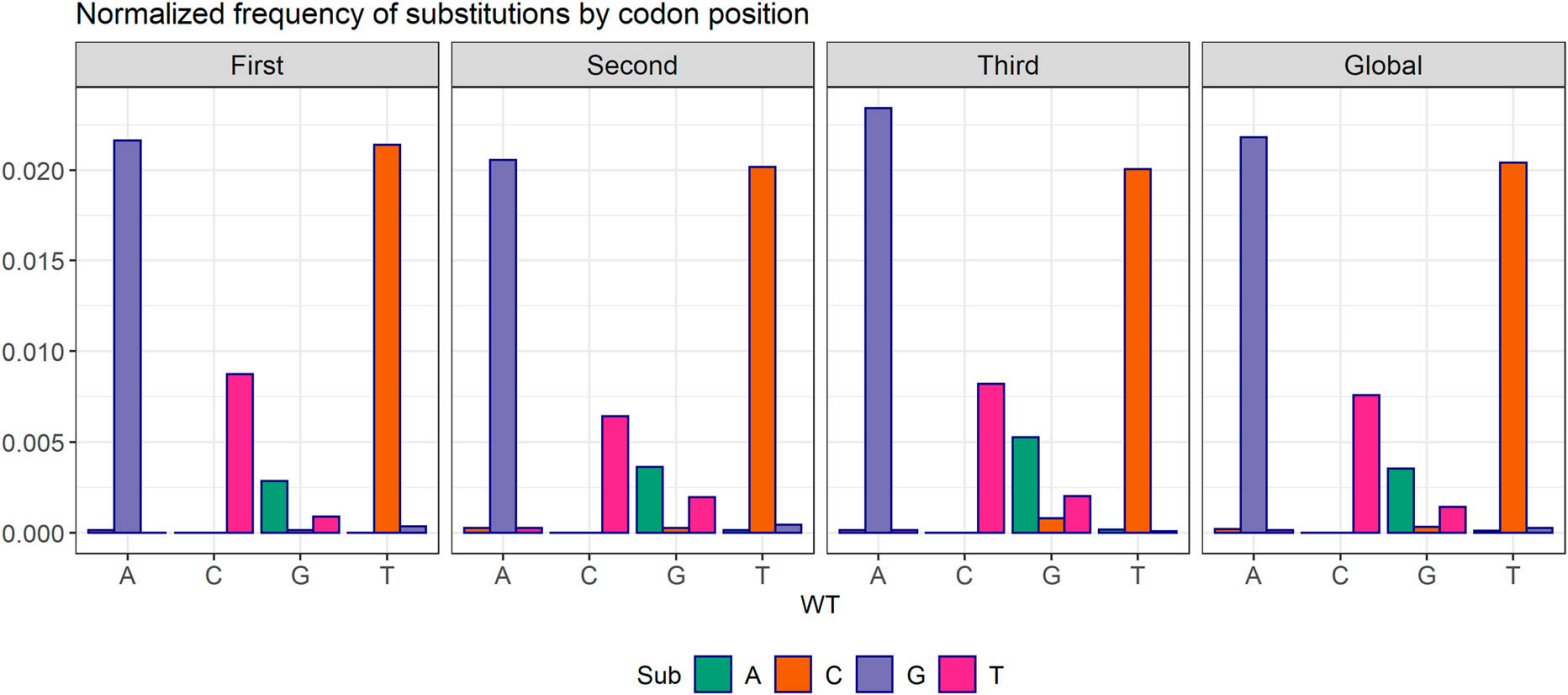
Changes observed between pairs of bases by codon position. In abscissas, the WT base, with variants in the bars. In ordinates, the ratio (#X → Y substitutions) / (#X sites). The pattern of substitutions is homogeneous in all three codon sites, despite the A → G and T → C biases observed. The incidence of transversions was very low, with G → T and T → A dominating.

Aggregation of all variants found in all patients by frequency bin (Table 3) and by substitution type yielded 642 polymorphic sites (16.8 8% of the 3822 bases in the *spike* gene), with 1090 SNVs. On determination of their abundance in the viral quasispecies, 6 positions had SNVs at ≥5% (Figure 1 and Supplementary Table S4). Of the 1090 SNVs identified, 942 (86.4%) comprised haplotypes present in <0.5% of the quasispecies. Among these, 1052 (96.5%) were transitions, mainly A → G and T → C (908, 83.3%) (Figure 2, Table 3). The SARS-CoV-2 *spike* gene is AT-rich (62.7%), containing 29.41% A and 33.25% T. However, 83.3% of all substitutions involved A → G and T → C changes (ADAR-type) showing a clear bias with respect to random substitutions. Non-synonymous substitutions dominated in all abundance bins except (5%, 10%]. The ratio of transitions to transversions and the ratio of ADAR-type to non-ADAR-type substitutions varied considerably as the abundance of SNVs increased, with values of 172.0 and 14.0, respectively, in the lowest frequency bin (0%, 0.2%], and 0.5 and 0.5, respectively, in the highest bin (10%, 50%] (Table 4). Only 6 SNVs were present at a frequency of >5% (Figure 1, panel A, Supplementary Table S4). The percentage of most SNVs was very low (<1%) (Figure 1, panel B). The fact that the ADAR-type to non-ADAR-type ratio was significantly higher in the lowest bins suggests that ADAR1 was the cause of those substitutions, with hypermutation effects, whereas the higher bins would incorporate the effects of haplotype fitness and selection (Table 4, Supplementary Figures S1 and S2).

**Table 3.** Counts of observed SNVs by frequency bins expressed in % ranges, among the 18 patients.

SNV	Frequency bins							Sum	%
	(0,0.2]	(0.2,0.5]	(0.5,1]	(1,2]	(2,5]	(5,10]	(10,50]		
A->C		2	1	1				4	0.37%
A->G	**160**	**243**	**20**	**10**	**7**	**1**		**441**	**40.46%**
A->T	1	1		1				3	0.28%
C->A									
C->G									
C->T	**15**	**51**	**17**	**12**	**3**	**1**		**99**	**9.08%**
G->A	**8**	**26**	**7**	**1**	**2**	**1**		**45**	**4.13%**
G->C		2		2				4	0.37%
G->T	2	5	2	5	2		2	18	1.65%
T->A		3						3	0.28%
T->C	**169**	**255**	**35**	**5**	**2**		**1**	**467**	**42.84%**
T->G		4	1	1				6	0.55%
Sum	355	592	83	38	16	3	3	1090	
%	32.57%	54.31%	7.61%	3.49%	1.47%	0.28%	0.28%		

The frequencies are as observed in the corresponding amplicon quasispecies. Transitions (in bold) accounted for 96.44% of observed SNVs. ADAR-type substitutions, A → G and T → C, accounted for 84.07% of the SNVs in this population. Most SNVs (86.92%) show abundances below 0.5% in the corresponding quasispecies.

**Table 4.** Ratio of transitions to transversions, ADAR-like substitutions to other substitution types, and synonymous to non-synonymous substitutions as observed by frequency bins.

	Frequency bins						
	(0,0.2]	(0.2,0.5]	(0.5,1]	(1,2]	(2,5]	(5,10]	(10,50]
Tr/Tv	172	33.2	26.0	2.70	7.00	Inf	0.50
ADAR/other	14	5.54	2.12	0.61	1.29	0.50	0.50
Syn/NonSyn	0.70	0.52	0.65	0.68	0.33	2.00	0.50

These results are obtained from Table 3.

Analysis of the mutations in relation to their position in the codon showed that the A → G and T → C transitions were randomly distributed and not restricted to specific positions (Figure 2). A significant overrepresentation of (A → G)/A relative to (G → A)/G, and of (T → C)/T relative to (C → T)/C, both overall and in each codon position, was confirmed by Fisher tests (Supplementary Table S6). The homogeneity of (A → G)/A substitutions with respect to (T → C)/T could not be rejected by Fisher test, which is consistent with the hypothesis that As are equally-likely edited on both strands (See Supplementary Table S7). Among the transversions, there was also a significant overrepresentation of (G → T)/G relative to (T → G)/T.

The analysis was completed by computing the following values for each patient: the number of bases in the respective consensus sequence, the number of polymorphic sites, the number of poly sites with A → G transitions, the number of poly sites with T → C transitions, and the corresponding fractions with respect to the total of sites, the total of A sites, and the total of T sites (Supplementary Table S8). Median values per patient were, respectively, 1124 A, 723 C, 704 G, 1271 T, 37 polymorphic sites (0.97%), 10 A → G (0.89% of A), and 12 T → C (0.94% of T). Overall there were 1,090 SNVs, 441 (40.5%) A → G transitions, and 467 (40.5%) T → C transitions.

On analysis of the number of substitutions in relation to their sites in the alignment of all 18 patients (Supplementary Table S9), we found 441 SNVs with A → G in any of the 230 sites with this type of substitution, and 467 SNVs with T → C in any of the 254 sites with this type of substitution. Conversely, there were 45 SNVs with G → A in any of the 41 sites with this type of substitution, and 99 SNVs with T → C in any of the 88 sites with this type of substitution. All the transversions observed were unique. In fact, most SNVs involved a single haplotype (Supplementary Table S3).

### Concurrent SNVs

Analysis of the quasispecies to detect SNVs common to more than one patient found that some variants were present in several patients, including one observed in 11. The mutations common to various patients showed no predilection for any specific region of the *spike*. The A2194G mutation was detected in 11/18 patients at a minimal abundance of 0.18% and a maximum of 0.26% (Table 5). All concurrent mutations were present at <1.0% (median [IQR] = 0.21% [0.19; 0.245]). Given the low abundance of these concurrent variants along with the very high abundance of the dominant haplotypes (>90%), we can assume that the former were generated independently in each patient during viral replication (Table 5). Moreover, all master sequences had abundances well above 50%, with most of them above 90%; hence, amplicon master sequences were equivalent to the amplicon quasispecies consensus sequences. Because of the high abundance of dominant haplotypes, we suggest that each patient was infected by a single haplotype (bottlenecking effect), and that all the variants observed had been generated independently within each patient.

**Table 5.** Concurrent SNVs.

Pos	WT	Var	*N*	Min Frq (%)	Max Frq (%)
2194	A	G	11	0.18	0.26
1389	T	C	10	0.18	0.23
2324	A	G	10	0.18	0.40
1683	T	C	9	0.18	0.23
1988	A	G	9	0.18	0.29
512	T	C	8	0.18	0.27
2092	T	C	8	0.20	0.28
3785	A	G	8	0.18	0.43
1224	A	G	7	0.19	0.26
1233	T	C	7	0.19	0.24
1616	T	C	7	0.18	0.32
2443	A	G	7	0.18	0.54
2942	T	C	7	0.22	0.55
467	A	G	6	0.18	0.34
885	T	C	6	0.18	0.27
1147	T	C	6	0.20	0.26
2479	A	G	6	0.20	0.26
2902	T	C	6	0.19	0.24
2913	T	C	6	0.18	0.24
2963	A	G	6	0.18	0.29
3298	A	G	6	0.19	0.24
3398	T	C	6	0.18	0.34
3452	A	G	6	0.18	0.28
3588	T	C	6	0.18	0.31
3779	A	G	6	0.19	0.30

Position, wild type base, variant base, number of patients with the substitution, and minimum and maximum frequency among all haplotypes showing the substitution. One SNV was seen in 11 patients, 2 were seen in 10 patients, 2 in 9 patients, 3 in 8 patients, 5 in 7 patients, 11 in 6 patients, as well as 13 in 5 patients and 25 in 4 patients that are not in the Table.

The level of concurrency may be better understood if we consider that overall there were 441 A → G substitutions in different haplotypes from the 18 patients, 436 when SNV duplicates in the same patient were removed, corresponding to 230 unique sites, with a ratio of 436/230 = 1.9. Furthermore, there were 467 T → C substitutions in different haplotypes, 460 when duplicates in the same patient were removed, corresponding to 254 unique sites, with a ratio of 460/254 = 1.8.

### Substitution context in the *spike* gene secondary structure

**Figure 3.**
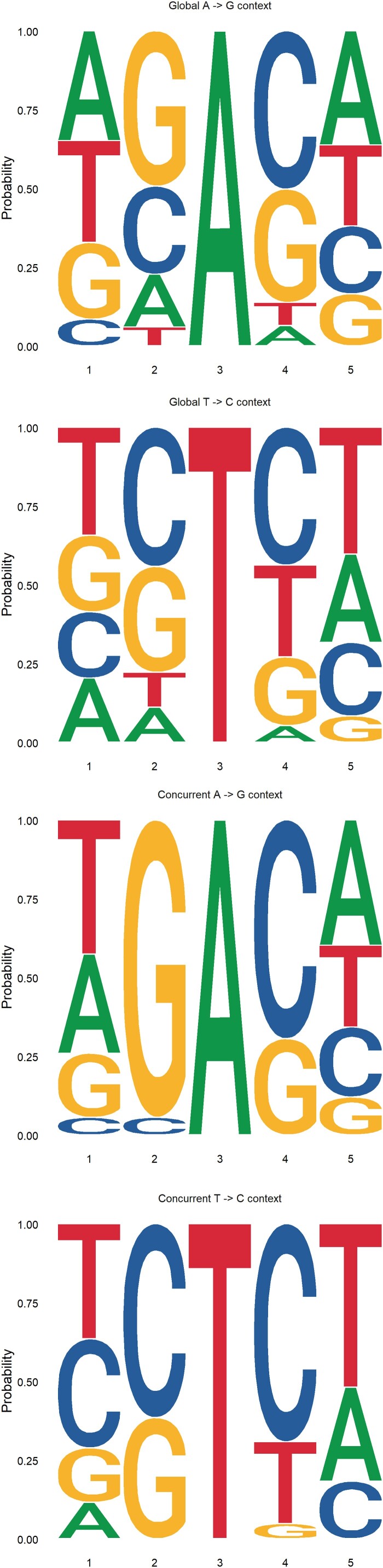
Location of concurrent substitutions occurring in more than 8 patients. A–D shows substitutions A to G and E–H shows substitutions T to C in the predicted complete *spike* RNA gene secondary structure of the positive strand in the wild-type (WT) genome.

In all 18 samples, A → G substitutions were studied to determine whether they were affected by neighbouring nucleotides; no specific pattern was detected at either the 5’ base (16.7% A, 6.0% T, 49.3% G, 28.0% C) or the 3’ base (6.4% A, 7.3% T, 36.0% G, 50.2% C). However, analysis of sites with recurrent mutations found in 5 or more patients showed a more specific pattern at the 5’ base (5.6% C, 94.4% G) and 3’ base (69.4% C, 30.6% G) (Figure 3).

**Figure 4.**
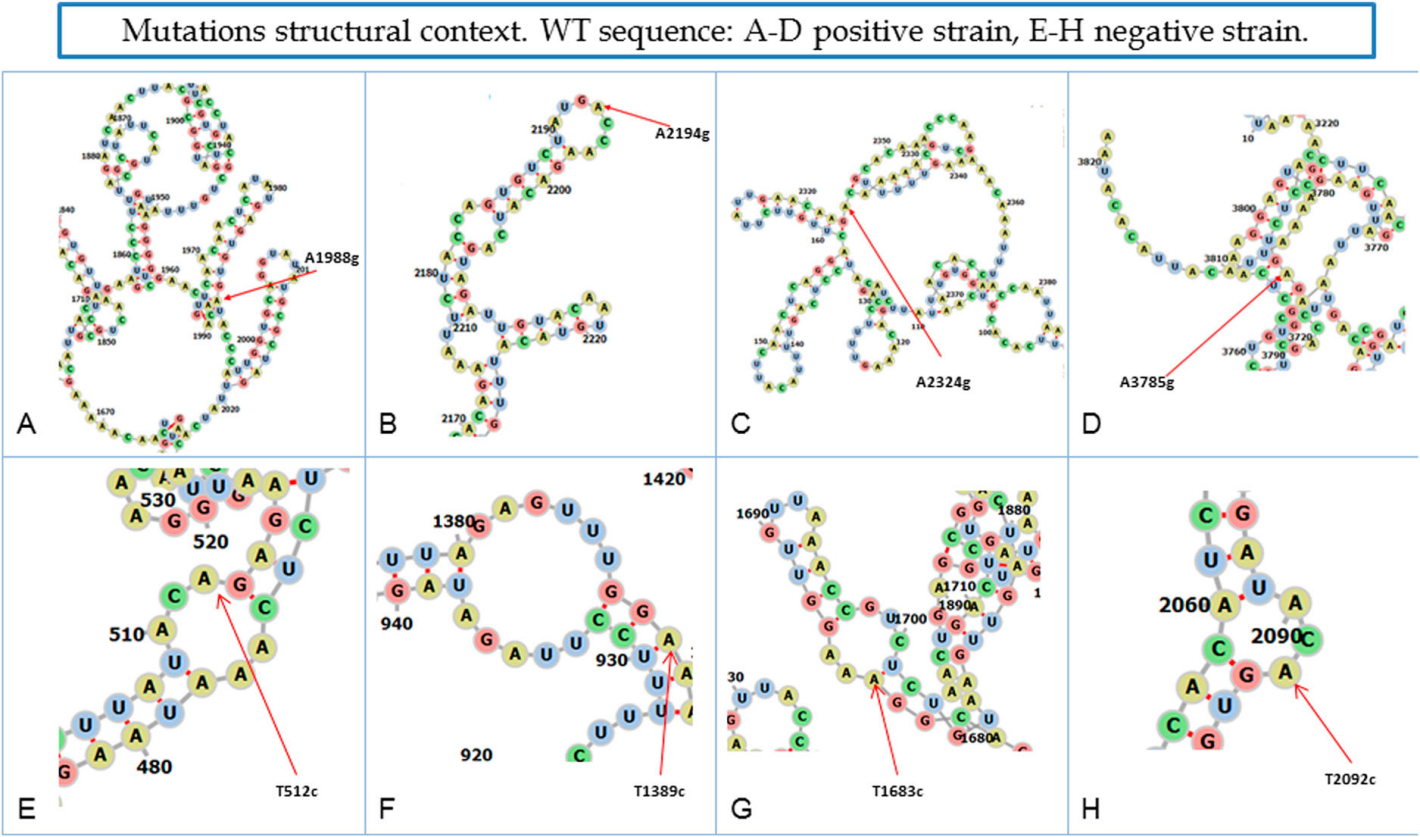
Sequence context of SARS-CoV-2 RNA edited sites. Top left includes all sites with A → G transitions. Top right includes sites with A → G concurrent transitions occurring in 4 or more patients. Bottom left includes all sites with T → C transitions. Bottom right includes sites with T → C concurrent transitions occurring in 4 or more patients.

Analysis of the substitution context of the RNA secondary structure only in SNVs common to at least 8 patients showed that the A → G transition in two substitutions (A1988G and A3785G) took place in the helix, one in the hairpin (A2194G), and one in a multibranched loop (A2324G) (Figure 4, panel A-D). In contrast, T → C changes occurred mainly in internal hairpins, with the exception of T1389C, which was found in the helix (Figure 4, panels E-H). None of the substitutions significantly changed the RNA secondary structure except for T1683C, which changed the internal loop into a predicted bulge (Supplementary Figure S3).

Inspection of the dinucleotides immediately before and after the A → G substitution sites showed a prominent 18.8% of AG and 19.5% of TG at 5’, and 25.7% of CA at 3’ compared to the randomly expected frequency of 6.25% (1/16). The same analysis on the T → C substitution sites also showed prominent dinucleotides: 19.8% TG, 14.1% CC, and 12.1% GC at 5’, and 18.9% CA, 17.4% TT, and 15.7% CT at 3’ (Supplementary Figure S4). This bias may be partially explained by the repetition of some substitutions among the patients studied.

### Fixed mutations worldwide

SNVs observed in 48,625 GISAID genomes worldwide by Mercatelli and Giorgi (2020) [40] were identified. Among the SNV types, we found that the fixed mutations were mainly C → T and G → T rather than ADAR1-associated changes (Supplementary Table S10).

## Discussion

Although SARS-CoV-2 RNA replication has a proofreading control, deletions and mutations have been reported in the *spike* gene [10]. NGS enables the deep study of the viral quasispecies and identification of mutations that might be selected and affect viral replication or pathogenesis. In this study, we analyzed samples from a group of COVID-19 patients with mild or severe disease by NGS at the start of the pandemic to investigate the complexity and variability of the viral quasispecies in the SARS-CoV-2 *spike* gene.

The variant analysis showed that all overlapping amplicons in the 18 patients had a prominent, highly abundant haplotype as well as a few haplotypes at low abundance bearing a single variant in most cases. Despite the deep analysis performed, no haplotype present in >0.8% of the quasispecies was found to be common to more than one patient. This finding supports the idea that only the most abundant haplotypes were transmitted person-to-person. Furthermore, it would indicate that there is a low probability that variants would be generated along the transmission chain. Instead, variant dominance would mainly occur in patients with a lengthy period of viremia.

The diversity analysis results suggest that SARS-CoV-2 quasispecies maturity and diversity are mainly driven by the duration of the infection, rather than by clinical severity. We found that SARS-CoV-2 is less diverse than other RNA viruses, such as HCV, HIV, and HBV, and that it makes use of mechanisms of variability other than its own RNA polymerase. The large coronavirus genome requires a balance between stability and diversity. The proofreading exoribonuclease 3’-5’ activity by nsp14 might have an impact on the high fidelity of replication [1, 41, 42]. However, viruses can exploit other sources of variation such as recombination [4] and acceptance of deletions [4–10] and insertions. In addition, they can use cellular mechanisms of innate immunity, such as ADAR1 editing activity [43]. Previous studies investigating the distribution of SARS-CoV-2 mutations [40, 44] are based on consensus sequences downloaded from the GISAID repository database [45, 46]; hence, viral genome subpopulations within the quasispecies of individual patients cannot be analyzed. Here we found that 86.9% of the variants observed using very deep sequencing (>70,000 reads) were present at <0.5%; hence, they can be considered rare variants. These rare variants appeared at the onset of nucleotide edition, prior to any fitness or fixation effects. Rare viral haplotypes may be more representative of the underlying mechanism causing the substitutions, whereas mutants observed at higher rates additionally represent the selected haplotypes with the highest fitness [47]. Accordingly, rare variants will mainly be transient because of their low abundance, low fitness value, or limited time to replicate and reach a significant load, unless a selective pressure (e.g. treatment with convalescent sera or antiviral monotherapy) occurs in patients with lengthy persistence of SARS-CoV-2 infection, such as immunocompromised individuals. In this situation, immune escape or resistant mutants may arise and be selected. This may have been the case of the British (VOC 202012/01), Brazilian (P.1 from B.1.1.28), Japanese (B.1.1.248), and South African (501Y.V2) variants, all sharing the N501Y mutation in the receptor-binding domain of the *spike* protein. Transmission of minority variants could, however, also occur in patients with lengthy infection and a high viral load, where there would be time enough for the virus to replicate and generate a highly fit variant, or for a variant to occur by chance after a bottlenecking event [48]. However, in the present study viral load did not correlate with COVID-19 disease severity. Severity has been linked with the patients’ demographic data, such as age and sex, and particularly with pre-existing comorbidities such as hypertension, diabetes, cardiovascular disease, chronic lung disease, and cancer, which have been associated with greater severity and a higher fatality rate. Furthermore, COVID-19 contributes to cardiovascular complications, including acute myocardial injury as a result of acute coronary syndrome, myocarditis, stress cardiomyopathy, arrhythmias, cardiogenic shock, and cardiac arrest [49, 50].

Most variants found (96.4%) were transitions, mainly A → G and T → C substitutions (88.1%), consistent with the action of ADAR1 [23]. ADARs edit specific sites or hyper-mutate full viral genes or genomes [51], which leads to mutations that can be selected depending on the dynamic interaction between the virus and host. Several viruses have used this genetic mechanism to generate variability that supports increases in infectivity and evolutionary potential [16–18, 43, 51, 52]. Various SNVs observed in this study were common to more than one patient, although at a low rate (<1%), suggesting that there are preferred nucleotide positions or “hot spots” within the genome where host enzymes such as ADAR1 can act. The probability that a particular point mutation would be found in multiple patients (hypothesis of independence) was almost negligible. Of note, all substitutions present in >3 patients were either A → G or T → C (at nt31 and 32, respectively), suggesting that ADAR1 had a role in generating them. It is important to highlight that the number of true substitutions should be even higher than the values reported in this study because of the very stringent filters used to avoid sequencing artifacts.

To determine whether ADAR1 editing is affected by the 5’ or 3’ neighbours of the substituted nucleotide [36], all A → G and T → C substitutions were analyzed, but no pattern emerged indicating a preferred neighbour on either the 5’ or 3’ end base. However, when only concurrent substitutions common to four or more patients were analyzed, the pattern was surprisingly more specific on both end bases. The 5’ neighbour preference of mutated A → G was G followed by C and A, whereas the 3’ neighbour preference of mutated T → C was C followed by T and G.

Neither the high A → G or T → C bias nor the neighbour preference led to the fixed mutations in the consensus genomes of the GISAID [45, 46]. Most recurrent substitutions occurring in four or more of our patients (51 of 63) could not be found in the GISAID genomes analyzed. Nine were found in a single GISAID genome, two were found in two GISAID genomes, and only one in three GISAID genomes. In our view, the most likely explanation for this would be related to the multiple non-specific editions (hypermutations) produced by ADAR. A small fraction of replicating genomes underwent ADAR edition and these sequences were edited several times, reducing the likelihood of replication fitness. That is why most ADAR-type substitutions were detected in genomes present at low rates (genomes with poor fitness) and why very few ADAR1-type substitutions were fixed and detected in consensus genomes (Supplementary Table S10). Thus, a limited ratio of available ADAR molecules to replicating genomes (stoichiometric reason), and a longer time of engagement of each ADAR molecule due to the large genome size, would limit the extension of ADAR1 hypermutation activity to a larger number of genomes, as has been observed in smaller viruses [53, 54]. Our results suggest that most fixed mutations would come from replication errors due to the polymerase, despite the proofreading mechanism.

The ADAR hypermutation hypothesis could be questioned by the fact that most haplotypes carried only a single substitution (Supplementary Table S4). Nonetheless, we should take into account that each amplicon was roughly a 1/13th of the *spike* gene, which in turn is the most variable region, but only about 10% of the full genome. Hence, if ADAR introduced only 2 substitutions on edited *spike* genes, it would imply around 20 mutations per genome, most of them non-synonymous (Table 4), which would suffice to impair the edited genomes. Regardless of the mechanism involved, the data suggest that ADAR has an antiviral action on the *spike* gene, although to a limited extent, as most of the replicating virions remained unaffected.

ADAR1 acts on dsRNA, and editing by this enzyme could occur in either the positive or the negative strand. ADAR1 editing of the positive strand during viral replication, in which dsRNA is formed, should lead to an A → I transition, whereas ADAR1 editing of the negative strand would result in a T → C transition, once the negative strand has served as the template for a new positive-strand [55]. Because of its considerable length, SARS-CoV-2 RNA has a complex secondary structure, with many double strands forming helices, hairpins, bulges, and loops [37, 38, 56]. Study of the *spike* secondary RNA structure in substitutions common to more than 8 patients showed that most mutations occurred in hairpins or loops. This suggests that the editing preferably occurred during replication. These results support the concept of multiple ADAR1 editions in a single genome, in either the positive or the negative strand, during RNA replication. Hypermutated genomes could carry deleterious mutations compromising fitness, and in a scenario of low selective pressure, these mutants would have a very low probability of being selected and transmitted. However, ADAR1 editing is a source of variation providing a reservoir of genomes that have a chance to be selected in response to an environmental challenge (e.g. immune response, vaccine, antiviral treatment), especially in long-term infected patients.

This study has some limitations, in particular, the small number of patients included, resulting from the need for deep sequencing of each sample, which generates an enormous amount of data. In addition, tissue samples from the patients studied were not available, and it was impossible to measure intracellular ADAR-1 activity. However, the findings open the door to investigation of whether new full variants of concern may arise because of the effects of ADAR1 on the quasispecies.

In conclusion, SARS-CoV-2 quasispecies variability was higher in patients with long-lasting infection, regardless of their severity status. Deep sequencing showed that most SARS-CoV-2 mutations present in the quasispecies were of the type produced by the action of ADAR1 editing enzymes, rather than substitutions inserted by the RNA-dependent RNA polymerase (nsp12). Furthermore, ADAR1 editing preferably occurred during replication (dsRNA with a+ and −strand), as the effects were similar on both strands. Nucleotide editing by ADAR1 took place on a small fraction of replicating genomes, but often resulted in multiple editions in the same genome, which can compromise genome fitness and replication. Further study at the long term showed very few ADAR-like mutations fixed in the GISAID consensus genomes, which is consistent with the idea that ADAR1 edition acts in the manner of a moderate antiviral by causing hypermutations that impair viral fitness. Finally, based on our data, we suggest that most, if not all, SARS-CoV-2 mutations that can be transmitted and become fixed in the consensus sequence are likely nsp12-induced and are produced in patients with high viremia and lengthy infection.

## Supplementary Material

TEMI_2021_0351_Gregori_et_al_Suppl_Figure_S4_editable.tiff

TEMI_2021_0351_Gregori_et_al_Suppl_Figure_S3_editable.tif

TEMI_2021_0351_Gregori_et_al_Suppl_Figure_S2_editable.tiff

TEMI_2021_0351_Gregori_et_al_Suppl_Figure_S1_editable.tiff

TEMI-2021-0351_Gregori_et_al_Suppl_Table_S10.xlsx

TEMI-2021-0351_Gregori_et_al_Suppl_Table_S9.xlsx

TEMI-2021-0351_Gregori_et_al_Suppl_Table_S8.xlsx

TEMI-2021-0351_Gregori_et_al_Suppl_Table_S7.xlsx

TEMI-2021-0351_Gregori_et_al_Suppl_Table_S6.xlsx

TEMI-2021-0351_Gregori_et_al_Suppl_Table_S5.xlsx

TEMI-2021-0351_Gregori_et_al_Suppl_Table_S4.xlsx

TEMI-2021-0351_Gregori_et_al_Suppl_Table_S3.xlsx

TEMI-2021-0351_Gregori_et_al_Suppl_Table_S2.xlsx

TEMI-2021-0351_Gregori_et_al_Suppl_Table_S1.xlsx

TEMI-2021-0351_Gregori_et_al_Legends_for_Suppl_Figures_S1_to_S4_and_Tables_S1_to_S10.docx
