# Supplementary figures and images for: Host-dependent editing of SARS-CoV-2 in COVID-19 patients
Source: Emerg Microbes Infect. 2021 Sep 5;10(1):1777–89. doi: 10.1080/22221751.2021.1969868 (PMC8425778; doi:10.1080/22221751.2021.1969868)

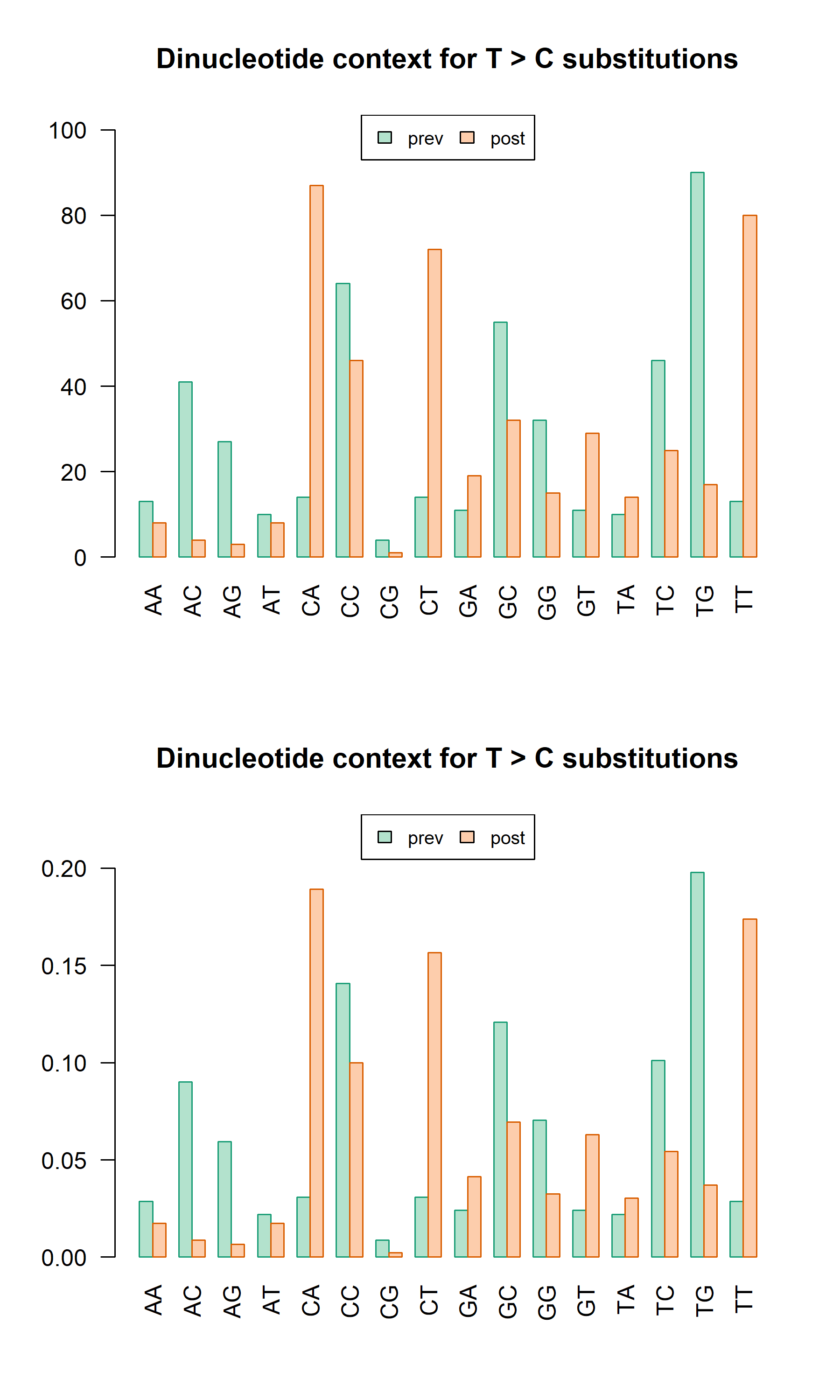

Supplement: TEMI_2021_0351_Gregori_et_al_Suppl_Figure_S4_editable.tiff [file TEMI_A_1969868_SM8282.tiff]

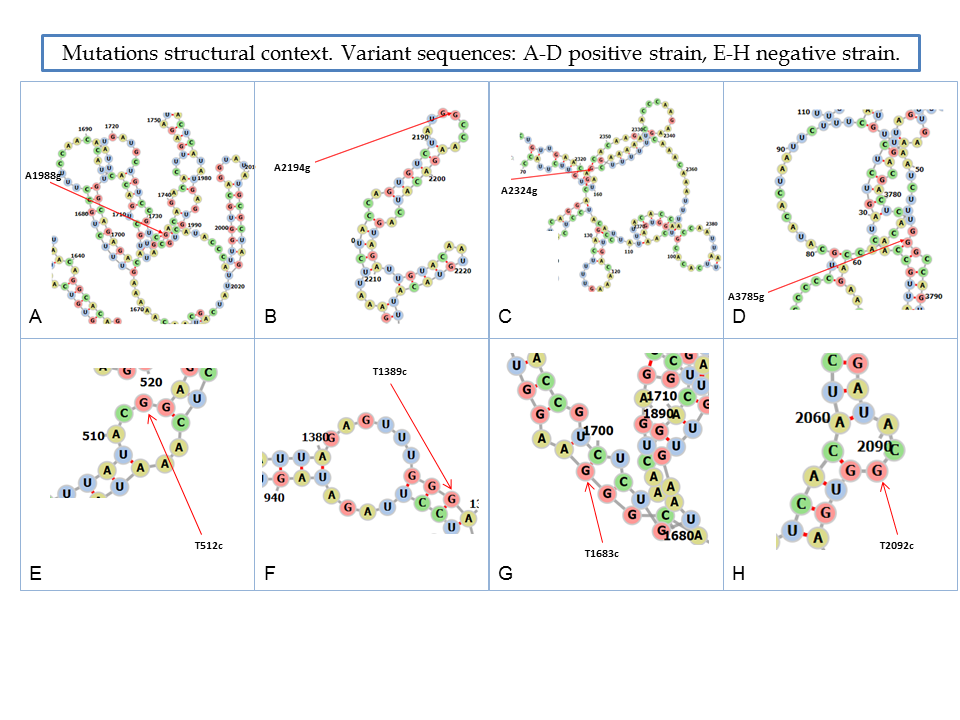

Supplement: TEMI_2021_0351_Gregori_et_al_Suppl_Figure_S3_editable.tif [file TEMI_A_1969868_SM8281.tif]

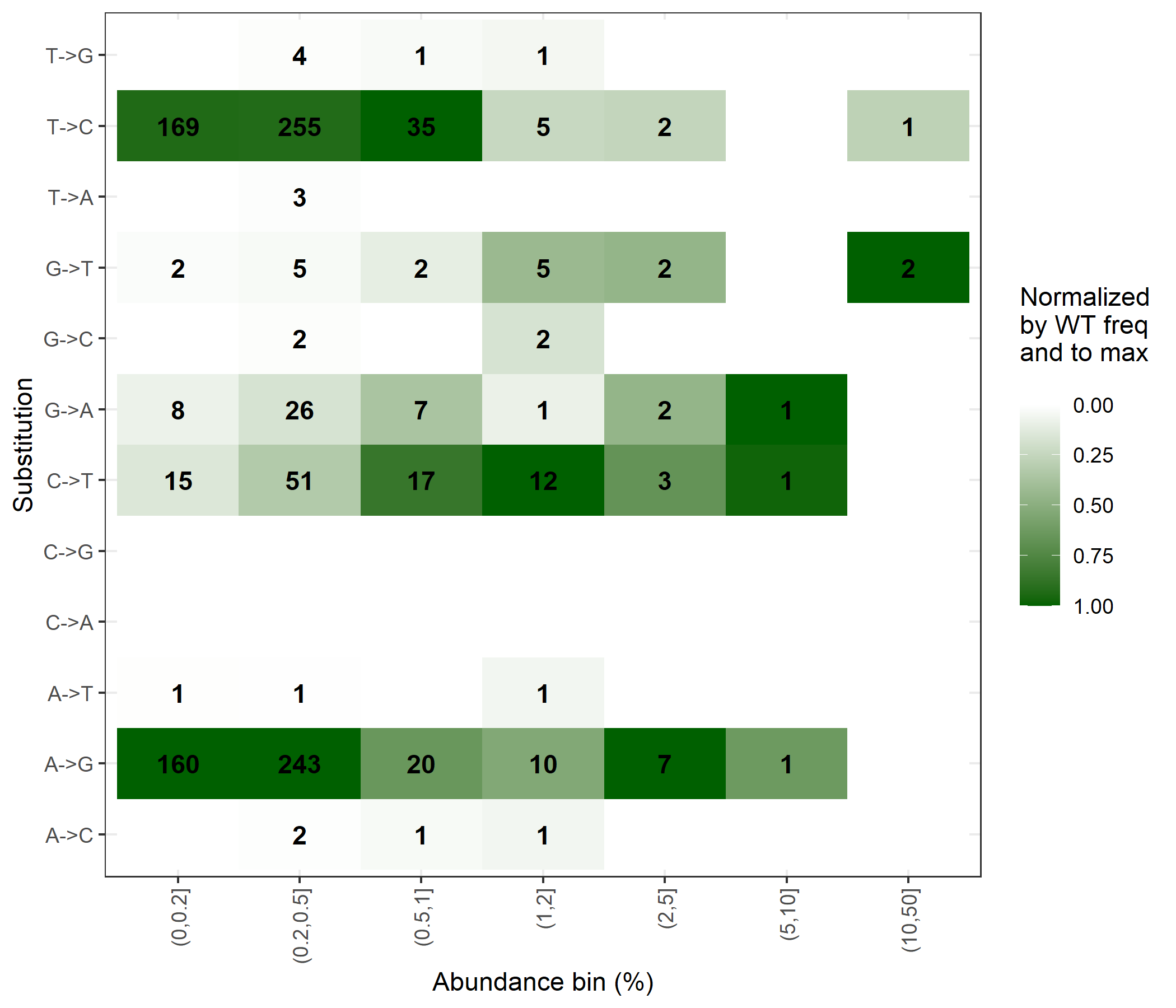

Supplement: TEMI_2021_0351_Gregori_et_al_Suppl_Figure_S2_editable.tiff [file TEMI_A_1969868_SM8280.tiff]

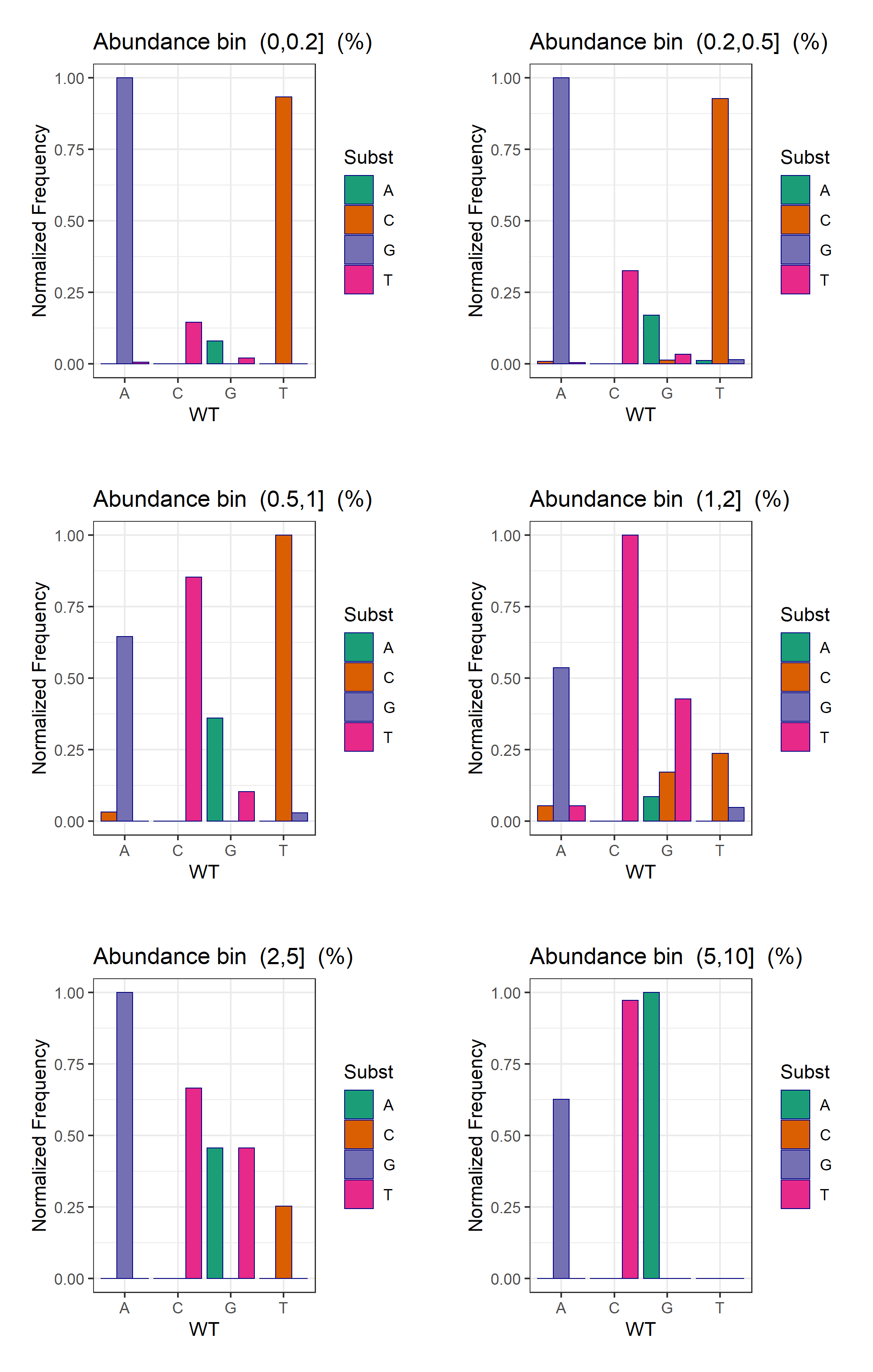

Supplement: TEMI_2021_0351_Gregori_et_al_Suppl_Figure_S1_editable.tiff [file TEMI_A_1969868_SM8279.tiff]
