# Supplementary material for: Host-dependent editing of SARS-CoV-2 in COVID-19 patients
Source: Emerg Microbes Infect. 2021 Sep 5;10(1):1777–89. doi: 10.1080/22221751.2021.1969868 (PMC8425778; doi:10.1080/22221751.2021.1969868)
Supplement: TEMI-2021-0351_Gregori_et_al_Legends_for_Suppl_Figures_S1_to_S4_and_Tables_S1_to_S10.docx [file TEMI_A_1969868_SM8268.docx]

**Supplementary Figure S1**: Barplots representing normalized frequencies of substitution types at different SNVs quasispecies abundance bins. The normalized frequency was computed in each plot by dividing the observed number of substitutions by the number of corresponding wild type nucleotides in the reference sequence (Wuhan), and then further divided by the max normalized frequency to obtain values in the 0-1 scale.

**Supplementary Figure S2**: Visualization of table 3B, with the number of SNVs by substitution type and categorized by abundance bin in %. The color scale provides normalized frequencies to the substitutions. These were computed by dividing the observed number of substitutions by the number of corresponding wild type nucleotides in the reference sequence (Wuhan); these normalized frequencies were further divided by the max normalized frequency in each bin.

**Supplementary Figure S3.** Location of substitutions occurring in more than 8 patients. A-D shows substitutions A to G and E-H shows substitutions T to C in the predicted complete spike RNA gene secondary structure of the positive strand in the mutated genome.

**Supplementary Figure S4**. Dinucleotide context in ADAR-like substitutions.

**Supplementary Table S1**: Number of amplicon-haplotypes observed by patient and amplicon.

**Supplementary Table S2**: Number of amplicon-haplotypes observed by patient (rows) categorized by abundance bin in % (columns).

**Supplementary Table S3**: Maximum number of mutations observed in any amplicon-haplotype with respect to the respective most abundant amplicon-haplotype (master sequence), by patient and amplicon.

**Supplementary Table S4**: Observed variants with respect to the corresponding quasispecies master sequence, and with abundances above 1%.

**Supplementary Table S5**: Number of quasispecies SNVs observed by patient (rows) categorized by abundance bin in % (columns).

**Supplementary Table S6**. Observed SNVs between pairs of bases by codon position and globally, and Fisher test p-values comparing complementary changes. There is a pronounced bias A → G with respect to G → A, and T → C with respect to C → T, in all codon positions and globally, affecting all observed transitions. In transversions the T → A in third codon position, and the G → T in second codon position show the same bias.

**Supplementary Table S7**. Homogeneity in the substitutions A → G and T → C

**Supplementary Table S8.** Counts by patient and globally of: nucleotides, number of polymorphic sites (Var) , number of A sites with G substitutions (A.G), number of T sites with C substitutions (T.C), fraction of sites which are polymorphic (% Var.st), fraction of A sites which have G substitutions (% A.G/A), fraction of T sites which have C substitutions (% T.C/T).

**Supplementary Table S9.** Count of substitutions by type. Unique: unique SNVs – 649 SNVs in 642 polymorphic sites. All: SNVs in the alignment of the 18 patients – 1,090 SNVs in 642 polymorphic sites.

**Supplementary Table S10**: All observed SNVs in 48,625 genomes from GISAID by Mercatelly & Giorgi, and unique SNVs observed in any position and any genome, provided that it is supported by 10 genomes at least. On the full genome, and on the gene S only.
